# When Combined with Pentamidine, Originally Ineffective Linezolid Becomes Active in Carbapenem-Resistant Enterobacteriaceae

**DOI:** 10.1128/spectrum.03138-22

**Published:** 2023-05-01

**Authors:** Miran Tang, Changrui Qian, Xiaotuan Zhang, Yan Liu, Wei Pan, Zhuocheng Yao, Weiliang Zeng, Chunquan Xu, Tieli Zhou

**Affiliations:** a Department of Clinical Laboratory, The First Affiliated Hospital of Wenzhou Medical University; Key Laboratory of Clinical Laboratory Diagnosis and Translational Research of Zhejiang Province, China; b School of Basic Medical Sciences, Wenzhou Medical University, Wenzhou, Zhejiang Province, China; c Department of Medical Lab Science, School of Laboratory Medicine and Life Science, Wenzhou Medical University, Wenzhou, Zhejiang Province, China; Innovations Therapeutiques et Resistances

**Keywords:** pentamidine, linezolid, carbapenem-resistant Enterobacteriaceae, combination, antimicrobial, antibiofilm, synergistic, efflux pump, outer membrane, membrane potential

## Abstract

The increasing prevalence of carbapenem-resistant Enterobacteriaceae (CRE) and their biofilm-relevant infections pose a threat to public health. The drug combination strategy provides a new treatment option for CRE infections. This study explored the synergistic antibacterial, antibiofilm activities as well as the *in vivo* efficacy against CRE of pentamidine combined with linezolid. This study further revealed the possible mechanisms underlying the synergy of the combination. The checkerboard and time-kill assays showed that pentamidine combined with linezolid had significant synergistic antibacterial effects against CRE strains (9/10). Toxicity assays on mammal cells (mouse RAW264.7 and red blood cells) and on Galleria mellonella confirmed that the concentrations of pentamidine and/or linezolid that were used were relatively safe. Antibiofilm activity detection via crystal violet staining, viable bacteria counts, and scanning electron microscopy demonstrated that the combination enhanced the inhibition of biofilm formation and the elimination of established biofilms. The G. mellonella infection model and mouse thigh infection model demonstrated the potential *in vivo* efficacy of the combination. In particular, a series of mechanistic experiments elucidated the possible mechanisms for the synergy in which pentamidine disrupts the outer membranes, dissipates the membrane potentials, and devitalizes the efflux pumps of CRE, thereby facilitating the intracellular accumulation of linezolid and reactive oxygen species (ROS), which ultimately kills the bacteria. Taken together, when combined with pentamidine, which acts as an outer membrane permeabilizer and as an efflux pump inhibitor, originally ineffective linezolid becomes active in CRE and exhibits excellent synergistic antibacterial and antibiofilm effects as well as a potential therapeutic effect *in vivo* on CRE-relevant infections.

**IMPORTANCE** The multidrug resistance and biofilm formation of Gram-negative bacteria (GNB) may lead to incurable “superbug” infections. Drug combinations, with the potential to augment the original treatment ranges of drugs, are alternative treatment strategies against GNB. In this study, the pentamidine-linezolid combination showed notable antibacterial and antibiofilm activity both *in vitro* and *in vivo* against the problem carbapenem-resistant Enterobacteriaceae (CRE). Pentamidine is often used as an antiprotozoal and antifungal agent, and linezolid is a defensive Gram-positive bacteria (GPB) antimicrobial. Their combination expands the treatment range to GNB. Hence, the pentamidine-linezolid pair may be an effective treatment for complex infections that are mixed by GPB, GNB, and even fungi. In terms of mechanism, pentamidine inhibited the outer membranes, membrane potentials, and efflux pumps of CRE. This might be a universal mechanism by which pentamidine, as an adjuvant, potentiates other drugs, similar to linezolid, thereby having synergistic antibacterial effects on CRE.

## INTRODUCTION

Carbapenems are used as defensive drugs to treat Gram-negative bacteria (GNB) infections (1). The alarmingly increasing prevalence of carbapenem-resistant Enterobacteriaceae (CRE) (1), combined with their strong biofilm formation, which renders more drug-resistant, recurrent, or even incurable infections (2), have significantly restricted the treatment options for their relevant infections (1, 3). Given the awkward situation of the development of new antimicrobials not keeping pace with the resistance of pathogens (4), more new, sustainable countermeasures, with the potential to expand the original treatment ranges of drugs (e.g., the rejuvenation of old antibiotics and drug combination therapy), are urgently needed to address CRE and their biofilm-associated infections. One powerful strategy out of these is treating GNB with antimicrobials that are usually restricted in Gram-positive bacteria (GPB) in combination with an antimicrobial adjuvant (5).

Linezolid exerts a strong antibacterial activity against GPB by inhibiting bacterial protein synthesis by binding to the rRNA of the 30S or 50S ribosomal subunits in the GPB (6). Although these intracellular targets of linezolid are also present in GNB (7), GNB is naturally resistant to linezolid because of its strong outer membrane barrier function, which is different from that of GPB, thereby rendering the drug unable to reach an effective intracytoplasmic concentration (8). Therefore, if this barrier effect is disrupted via combination with a certain membrane inhibitor (8), linezolid may enter into GNB and exert its antibacterial activity against it. In a recent study, the antibacterial effect of a colistin-linezolid combination was demonstrated in Pseudomonas aeruginosa (P. aeruginosa), Escherichia coli (E. coli), and Acinetobacter baumannii (A. baumannii) (9). A possible mechanism has also been revealed; colistin increases the intracellular accumulation of linezolid in GNB by disrupting the permeability barrier of the outer membrane and inhibiting the activity of efflux pumps (7).

Pentamidine, which was originally used as an antiprotozoal and antifungal agent (10), has recently been studied as an antimicrobial adjuvant (4, 5, 11–13). Pentamidine combined with rifampin, novobiocin, and erythromycin exhibited synergistic activity against a wide range of GNB (5). In addition, pentamidine, in combination with aminoglycosides, tigecycline, and doripenem, showed potent *in vitro* activity against carbapenem-resistant and/or colistin-resistant E. coli, Klebsiella pneumoniae (K. pneumoniae), and Enterobacter cloacae (E. cloacae) (12). Unfortunately, none of these studies involved the antibiofilm activity of these drug pairs. Further, to our knowledge, there have been no reports thus far that have introduced the combination of pentamidine and linezolid for the treatment of CRE, let alone their antibiofilm activity, potential *in vivo* efficacy, and synergy mechanisms.

This study aimed to assess the synergistic antibacterial and antibiofilm activities *in vitro* and *in vivo* against CRE as well as the potential mechanisms for the synergy of a pentamidine-linezolid combination.

## RESULTS

### Bacterial strains and antimicrobial susceptibility profiles.

A total of 10 nonduplicated Gram-negative clinical isolates were selected, including 4 E. coli, 3 K. pneumoniae, and 3 E. cloacae. The MIC_90_ (i.e., the minimum inhibitory concentration [MIC] value that 90% of isolates are at or below) values of antimicrobial agents against the 10 isolates are shown in Table 1. All of the 10 strains were defined as CREs, as they were resistant to at least one of the carbapenem agent, and E. coli DC3737 was defined as carbapenem and colistin-coresistant.

**TABLE 1:** Antimicrobial susceptibility profiles of the studied clinical CRE isolates^*a*^

Antimicrobial agents		AMP	CRO	CAZ	FEP	ATM	IMP	MEM	ETP	CIP	LVX	GEN	TOB	AMK	COL	PNT	LNZ
Breakpoints (S-R)		8–32	1–4	4–16	2–16	4–16	1–4	1–4	0.5–2	0.25–1	0.5–2	4–16	4–16	16–64	2	NA	NA
**Species**	**Isolate**	**MIC (mg/L)**															
K. pneumoniae	FK7921	>32	≥64	≥64	≥64	≥64	8	8	16	≤4	≤2	≤1	≤1	≤2	0.25	512	512
	FK7978	>32	≥64	≥64	≥64	≥64	8	8	16	≤4	≤2	≤1	≤1	≤2	0.25	1,024	>1,024
	FK7018	>32	≥64	≥64	≥64	≥64	8	8	32	≥4	8	≥16	1	≤2	0.25	2,048	>1,024
E. cloacae	CG1779	>32	≥64	≥64	≥64	≥64	8	8	16	≥4	≥8	≤1	≤1	≤2	0.25	2,048	>1,024
	CG1591	>32	≥64	≥64	≥64	≥64	4	4	16	≥4	≥8	≥16	8	≤2	0.25	1,024	>1,024
	CG1781	>32	≥64	≥64	≥64	≥64	4	8	16	≥4	≥8	≤1	≤1	≤2	0.25	2,048	1,024
E. coli	DC6581	>32	>64	>64	≥64	≥64	8	4	16	≥4	≥8	≥16	≥16	8	0.125	256	512
	DC7143	>32	>64	>64	≥64	≥64	4	4	8	≥4	≥8	≤1	≤1	≤2	0.25	256	512
	DC6669	>32	>64	>64	≥64	≥64	4	4	8	≥4	≥8	≥16	8	≤2	0.25	256	>1,024
	DC3737	>32	>64	>64	≥64	≥64	8	16	16	≥4	≥8	≥16	≥16	16	16	64	512

a AMP, ampicillin; CRO, ceftriaxone; CAZ, ceftazidime; FEP, cefepime; ATM, aztreonam; IMP, imipenem; MEM, meropenem; ETP, ertapenem; CIP, ciprofloxacin; LVX, levofloxacin; GEN, gentamicin; TOB, tobramycin; AMK, amikacin; COL, polymyxin; PNT, pentamidine; LNZ, linezolid. S, susceptible; R, resistant. NA, not applicable. MIC, minimum inhibitory concentration.

### Checkerboard assays.

**FIG 1:**
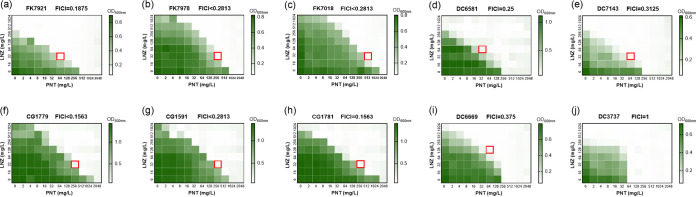
Heat map of the checkerboards between pentamidine and linezolid against the 10 CRE clinical strains: 3 K. pneumoniae (a–c), 4 E. coli (d, e, i, and j), and 3 E. cloacae (f–h). Dark regions represent higher cell densities. Red boxes indicate the selected combination concentrations of linezolid and pentamidine when they showed synergistic activity. The fractional inhibitory concentration index (FICI) shown in each isolate is calculated either according to the MIC of the linezolid and pentamidine combination or alone, as indicated in the red boxes. PNT, pentamidine; LNZ, linezolid.

Fig. 1 revealed the interaction patterns of pentamidine-linezolid combinations on 10 clinical CRE isolates. The combination of pentamidine with linezolid showed synergistic activity against 9 of the 10 CRE strains (fractional inhibitory concentration index (FICI) < 0.5) (Fig. 1A–I), except for the carbapenem and colistin-coresistant E. coli DC3737 (FICI = 1) (Fig. 1J). The red boxes indicate the selected combination and the corresponding, respective single-agent concentrations in each bacteria case throughout the study. The bottom rows and the leftmost columns show that the MICs of pentamidine and linezolid against these CRE isolates ranged from 256 to 2048 mg/L and from 512 to ≥1024 mg/L, respectively. When combined with pentamidine, the MIC values for linezolid were significantly decreased by 8 to >64 times. The checkerboards showed that the combination of the two drugs had significant antibacterial effects against CRE.

### Safety evaluation of the drugs and their concentrations.

**FIG 2:**
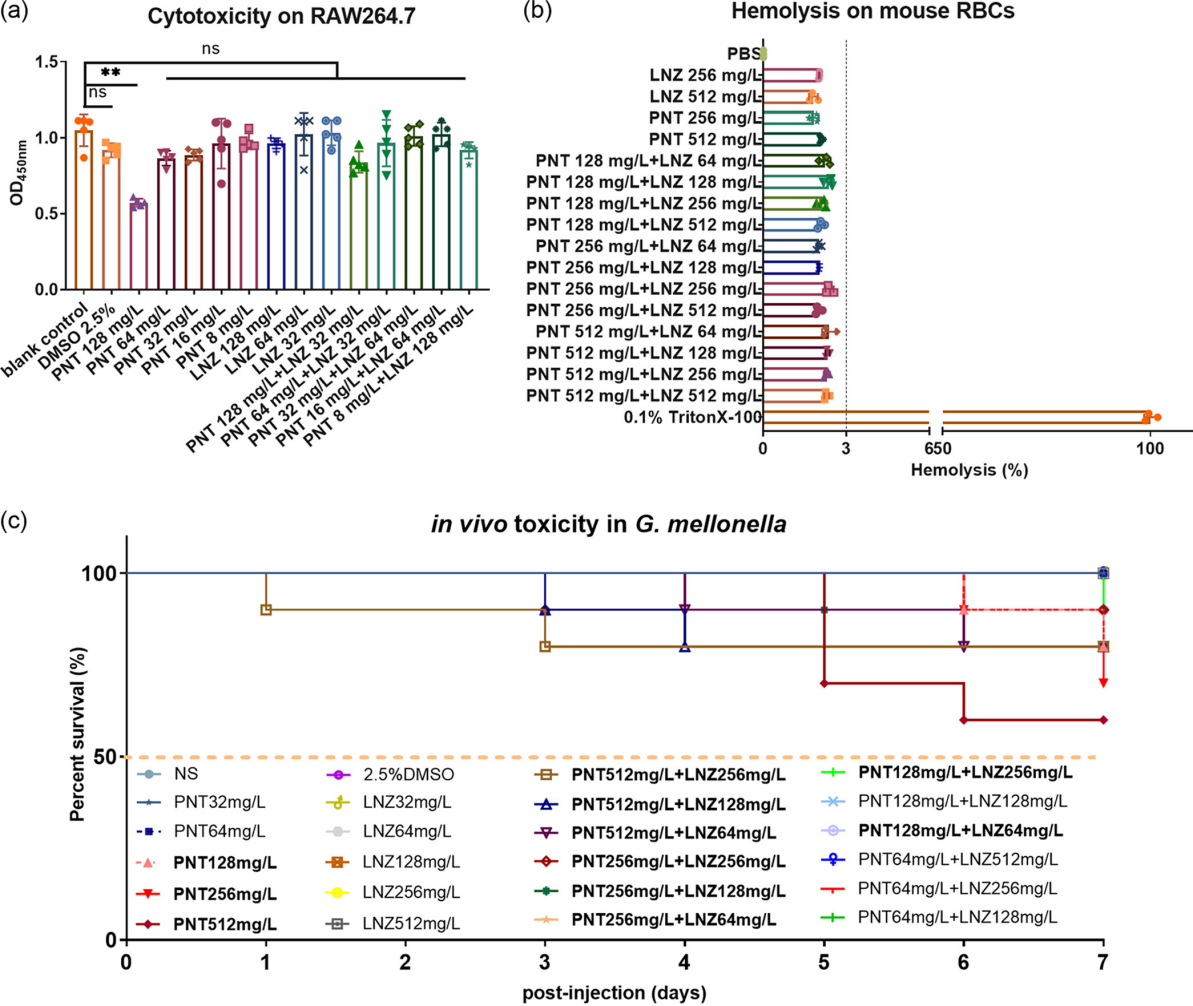
Assessment of the safety of the studied drugs and their concentrations. (A) The cytotoxicity effects of pentamidine, linezolid, pentamidine-linezolid pairs with different concentrations, and 2.5% dimethyl sulfoxide (DMSO) were tested against the RAW 264.7 murine macrophage cell line using the Cell Counting Kit-8 (CCK-8) method (absorbance values at 450 nm). The data are expressed as the mean ± standard deviation from five biological replicates. ns, not statistically significant; **, *P *< 0.01. (B) Hemolytic activities of pentamidine with different concentrations. A hemolysis rate of lower than 5% was considered to have no hemolytic activity. All of the hemolysis rates of the experimental groups were lower than 3% (marked with a dotted line). (C) The *in vivo* toxicity effects of pentamidine, linezolid, and pentamidine-linezolid pairs with different concentrations were further confirmed in G. mellonella. The critical line of LD_50_ is marked with a dotted line. Drug concentrations in bold indicate that there was the death of larvae within 7 days, whereas the others in regular type indicate a lack of death. All of the tested drug concentrations (32 to 512 mg/L pentamidine and 32 to 512 mg/L linezolid alone or in combination) were lower than the LD_50_ value. PNT, pentamidine; LNZ, linezolid.

The safety of the selected combination and the corresponding, respective single-agent concentrations that are indicated in the red boxes in Fig. 1 was evaluated using a Cell Counting Kit-8 (CCK-8) cytotoxicity test on RAW264.7 cells, a hemolysis of red blood cells (RBCs) assay, and a LD_50_ (50% lethal dose) test on G. mellonella larvae. In addition to the 128 mg/L pentamidine (*P *< 0.01), pentamidine monotherapy (8, 16, 32, and 64 mg/L), linezolid monotherapy (32, 64, and 128 mg/L), and pentamidine-linezolid pairs (8-128, 16-64, 32-64, 64-32, 128-32 mg/L) were not toxic to the cells (*P *> 0.05) (Fig. 2a). We further investigated the toxic effects of various concentrations of drugs on mouse RBCs. As shown in Fig. 2b, when the concentrations of pentamidine and linezolid, alone or in combination, reached 512 mg/L, the hemolysis rate was still less than 3%, and no hemolysis was observed by the naked eye. Through an *in vivo* toxicity test, we found that the mortality of G. mellonella larvae at 7 days postinjection with pentamidine (32 to 512 mg/L) and linezolid (32-512 mg/L), alone or in combination, were lower than 50%. In other words, 32 to 512 mg/L pentamidine and 32 to 512 mg/L linezolid, alone or in combination, at a dose that was lower than the LD_50_, were not considered to be toxic *in vivo* (Fig. 2c). Taken together, all of the concentrations of the drugs to which we referred were relatively safe for further study.

### Time-kill assay.

**FIG 3:**
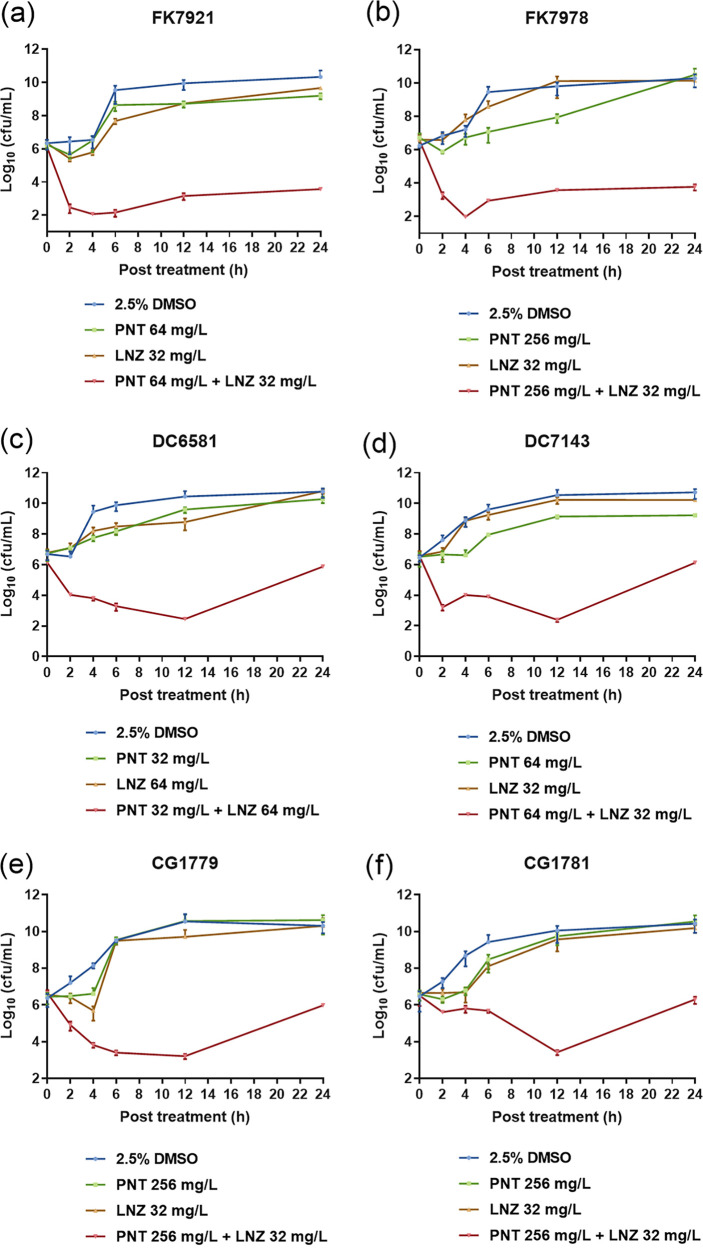
Time-kill kinetics of pentamidine monotherapy, linezolid monotherapy, and combination therapy against six randomly selected clinical CRE strains: two K. pneumoniae (a and b), two E. coli (c and d), and two E. cloacae (e and f). The data are expressed as the mean ± standard deviation from three biological replicates. PNT, pentamidine; LNZ, linezolid.

To further confirm the synergy of the combination, a time-kill assay was conducted to study the effect of the combination on the growth kinetics of CRE in six randomly selected CRE isolates (two K. pneumoniae, two E. coli, and two E. cloacae). The combination concentrations showing FICI < 0.5 and their corresponding single-agent concentrations against each strain from the checkerboard assay (red boxes in Fig. 1) were used for the time-kill assay. Fig. 3 shows the time-kill profiles for pentamidine and linezolid, alone and in combination, against 6 randomly selected CRE isolates (two K. pneumoniae, two E. coli, and two E. cloacae). The overall trends of the six CRE isolates among the control, pentamidine monotherapy, linezolid monotherapy, and combination groups were similar. Bacteria that were exposed to pentamidine or linezolid alone displayed comparable killing curves to those of the control cultures without drug treatment. In contrast, their combination resulted in a much more rapid bacterial killing trend, showing a dramatic decrease of 2 to 4 log_10_ CFU/mL of viable cells, compared with the initial bacterial inoculum within 12 h, which revealed a remarkable bactericidal activity of the combination of pentamidine and linezolid. Although there were bacterial regrowths within 12 to 24 h in the E. coli (Fig. 3c and d) and E. cloacae (Fig. 3e and f) strains, the CFU/mL in the combination groups were 2 to 6 log_10_ lower than those in the monotherapy control groups at 24 h in all of the tested strains, implying that the combination has good synergistic bacteriostatic effects against CRE.

### Inhibitory and eradicating effects on CRE biofilm.

**FIG 4:**
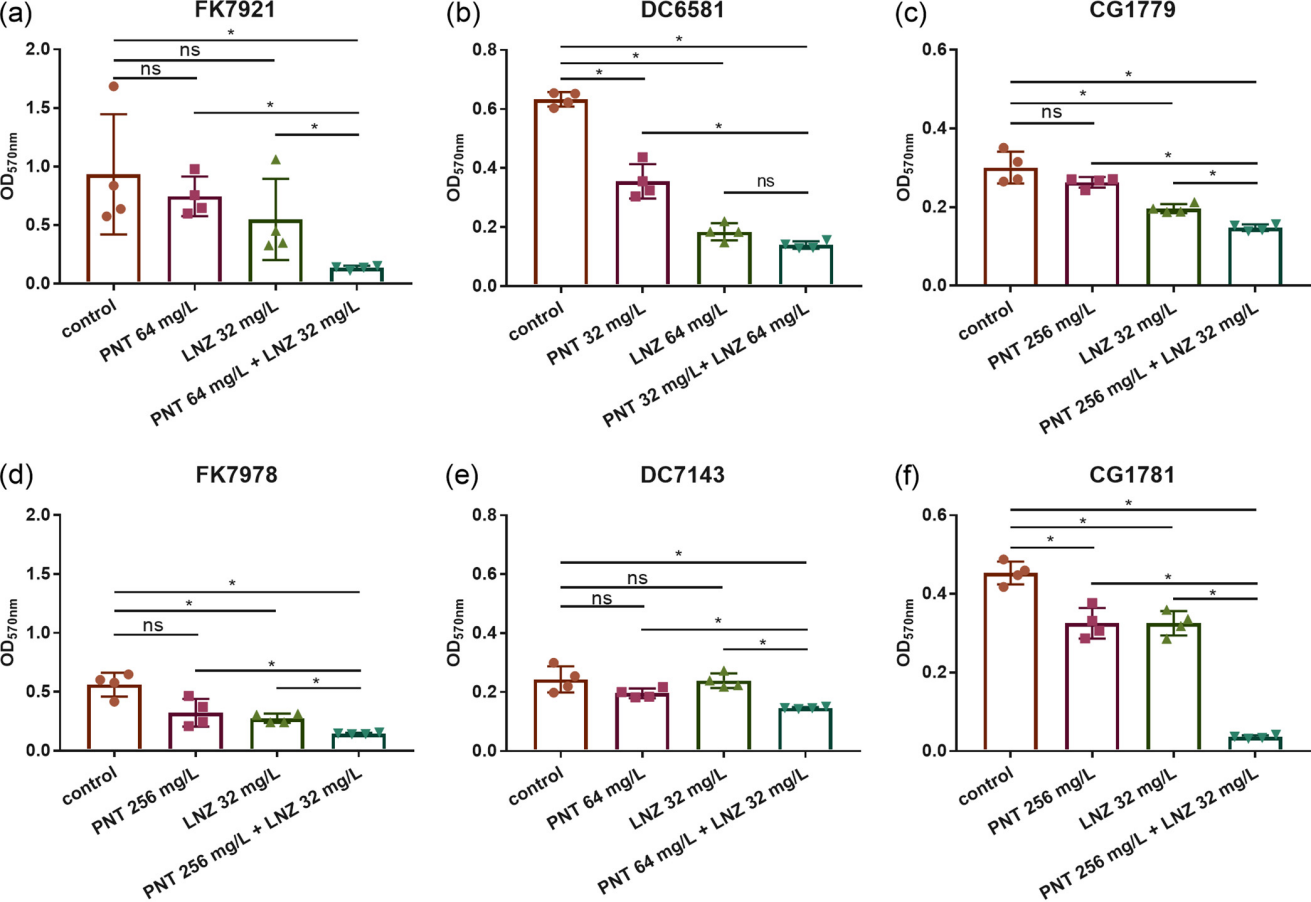
Biofilm formation inhibition for pentamidine in combination with linezolid against six clinical CRE strains: two K. pneumoniae (a and d), two E. coli (b and e), and two E. cloacae (c and f). The combination concentrations showing FICI < 0.5 and their corresponding single-agent concentrations against each strain were accordingly selected from the checkerboard assay (red boxes in Fig. 1) as the experimental groups, whereas the control groups were without drug treatments. The data are expressed as the mean ± standard deviation from four biological replicates. ns, not statistically significant; *, *P *< 0.05, as analyzed via a Student’s *t* test. PNT, pentamidine; LNZ, linezolid.

**FIG 5:**
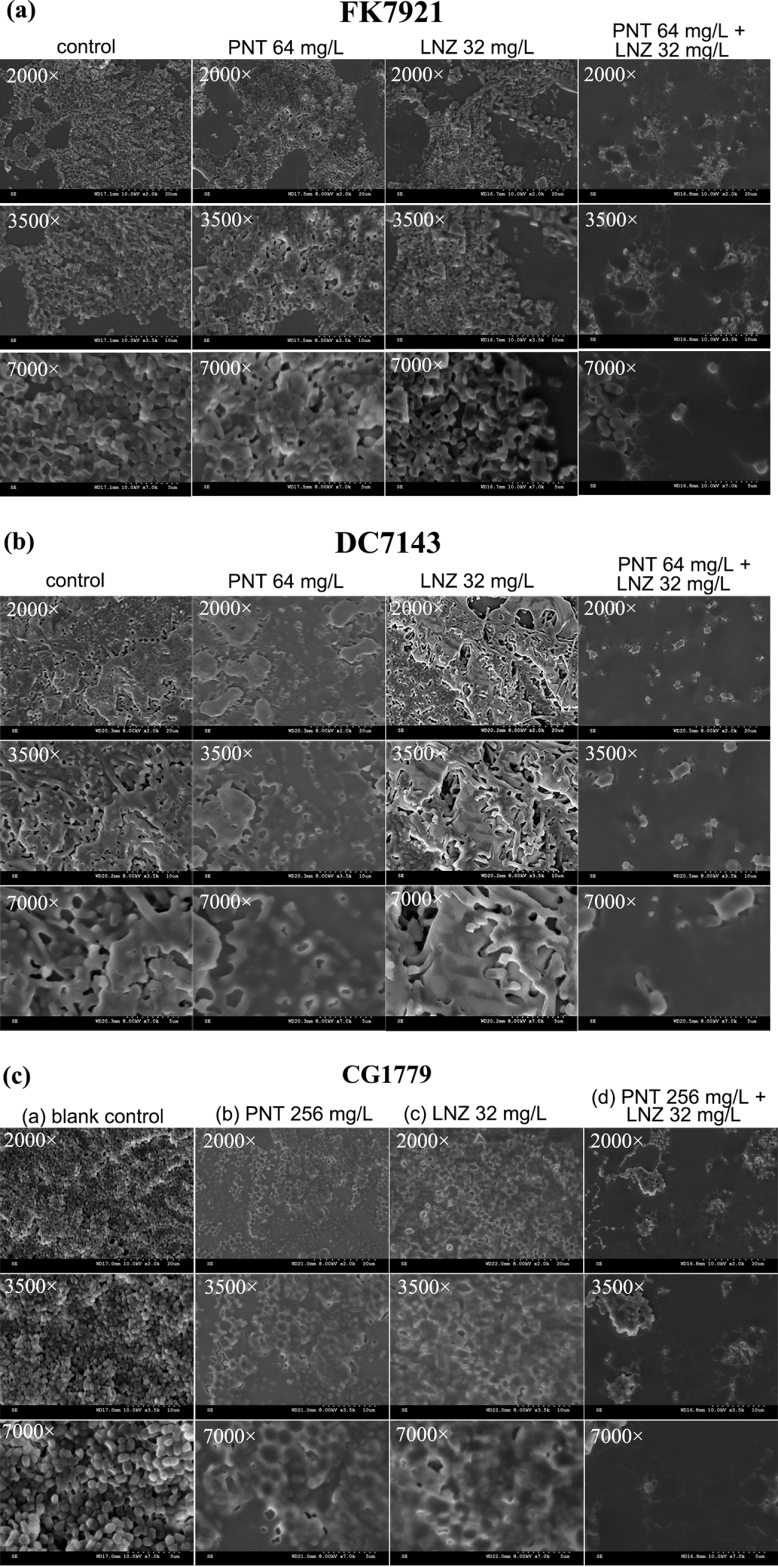
SEM images of biofilms of three clinical CRE strains, namely, K. pneumoniae FK7921 (a), E. coli DC7143 (b), and E. cloacae CG1779 (c), treated with or without pentamidine, linezolid, or both. The synergistic combination concentrations and the corresponding, respective single-agent concentrations, as per the checkerboard experiment (red boxes in Fig. 1), were selected as the experimental groups, whereas the control groups were without drug treatments. 3 images were acquired from the same field of the same group, with 2,000×, 3,500×, and 7,000× magnifications. PNT, pentamidine; LNZ, linezolid.

The same six clinical CRE strains that were used in the time-kill experiments were used to detect the inhibitory effects on the biofilm formation of the combination via crystal violet (CV) staining (Fig. 4). Pentamidine combined with linezolid, at the concentration equivalent to the 1× MIC combination, inhibited biofilm formation in all of the tested strains, compared to the control group (*P *< 0.05) (Fig. 4a–f). Additionally, biofilm formation was inhibited by the drug combination, compared with the monotherapy groups in five strains (*P *< 0.05) (Fig. 4a, c–f), with the exception of E. coli DC6581, in which there was no significant difference between the combination and the linezolid monotherapy groups (*P *> 0.05) (Fig. 4b). To further characterize the inhibitory effects of the combination on biofilm formation, scanning electron microscope (SEM) images were collected (Fig. 5). Fig. 5 shows SEM images of biofilms of three representative bacteria (K. pneumoniae FK7921 [Fig. 5a], E. coli DC7143 [Fig. 5b], and E. cloacae CG1779 [Fig. 5c]) that were treated with or without pentamidine, linezolid, or both. As for any one of the tested strains, bacterial cells without treatment or treated with either pentamidine or linezolid alone formed abundant and thick biofilms that were constructed with rich cell masses that were interwoven into a completely dense, membrane-like mesh. Monotherapy had no significant impact on the overall morphology of the bacterial biofilms, compared to the control groups (without drugs). However, in combination, pentamidine-linezolid resulted in a significant decrease in cells and disruptions in membrane-like structures. So, we can conclude that the pentamidine-linezolid pair has good synergistic inhibition effects against CRE biofilms.

**FIG 6:**
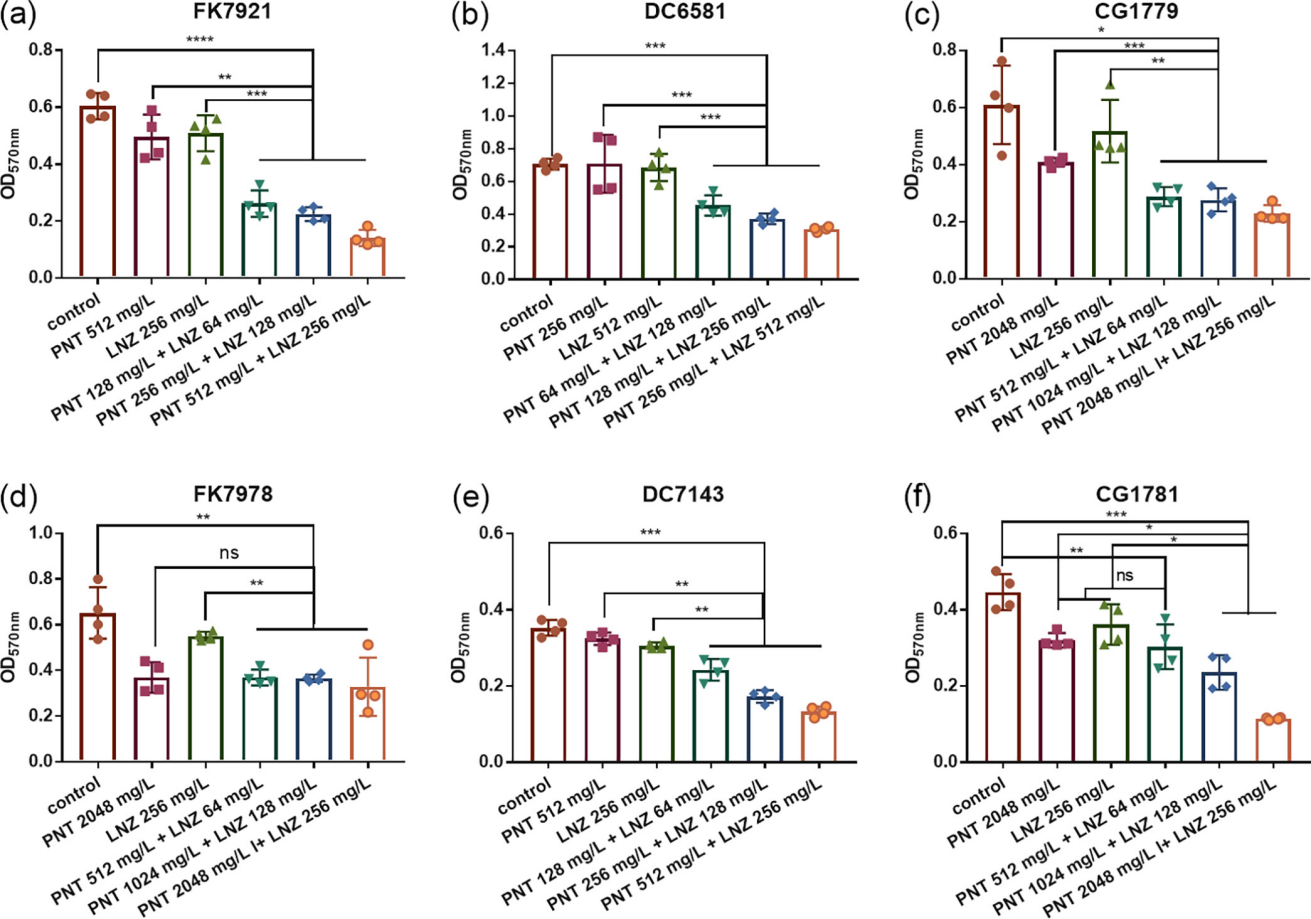
Eradication of preformed biofilms for pentamidine in combination with linezolid against six clinical CRE strains: two K. pneumoniae (a and d), two E. coli (b and e), and two E. cloacae (c and f). 2×, 4×, and 8× of the combination concentrations showing FICI < 0.5 and their corresponding single-agent concentrations against each strain, as per the checkerboard assay (red boxes in Fig. 1), were accordingly selected as the experimental groups, whereas the control groups were without drug treatments. The data are expressed as the mean ± standard deviation from four biological replicates. ns, not statistically significant; *, *P *< 0.05; **, *P *< 0.01; ***, *P *< 0.001; ****, *P *< 0.0001, as analyzed via a Student’s *t* test. PNT, pentamidine; LNZ, linezolid.

The study also investigated the eradication efficacy of the combination against the preformed biofilms of the six clinical CRE strains that were selected in the biofilm inhibition assay above. Fig. 6 shows that pentamidine combined with linezolid eradicated the biofilms of all the strains, compared with the control groups (*P *< 0.05). To further validate whether a pentamidine-linezolid combination could eradicate preformed mature biofilms more significantly than could monotherapy, the biofilm mass at the 2×, 4×, and 8× combination concentrations were compared with that at the respective single-agent concentration corresponding to the 8× combined concentration. The results showed that, except for K. pneumoniae FK7978 (Fig. 6d) and E. cloacae CG1781 (Fig. 6f), 2×, 4×, and 8× MIC in combination could eradicate preformed mature biofilms more significantly than could the pentamidine or linezolid monotherapy equivalents to the 8× MIC combination in the other four isolates (*P *< 0.05) (Fig. 6a–c, e). It can be concluded that the combination of pentamidine and linezolid can eradicate the preformed biofilms of CRE to a certain extent, and the more pronounced effects were obtained by using the higher combination dose.

**FIG 7:**
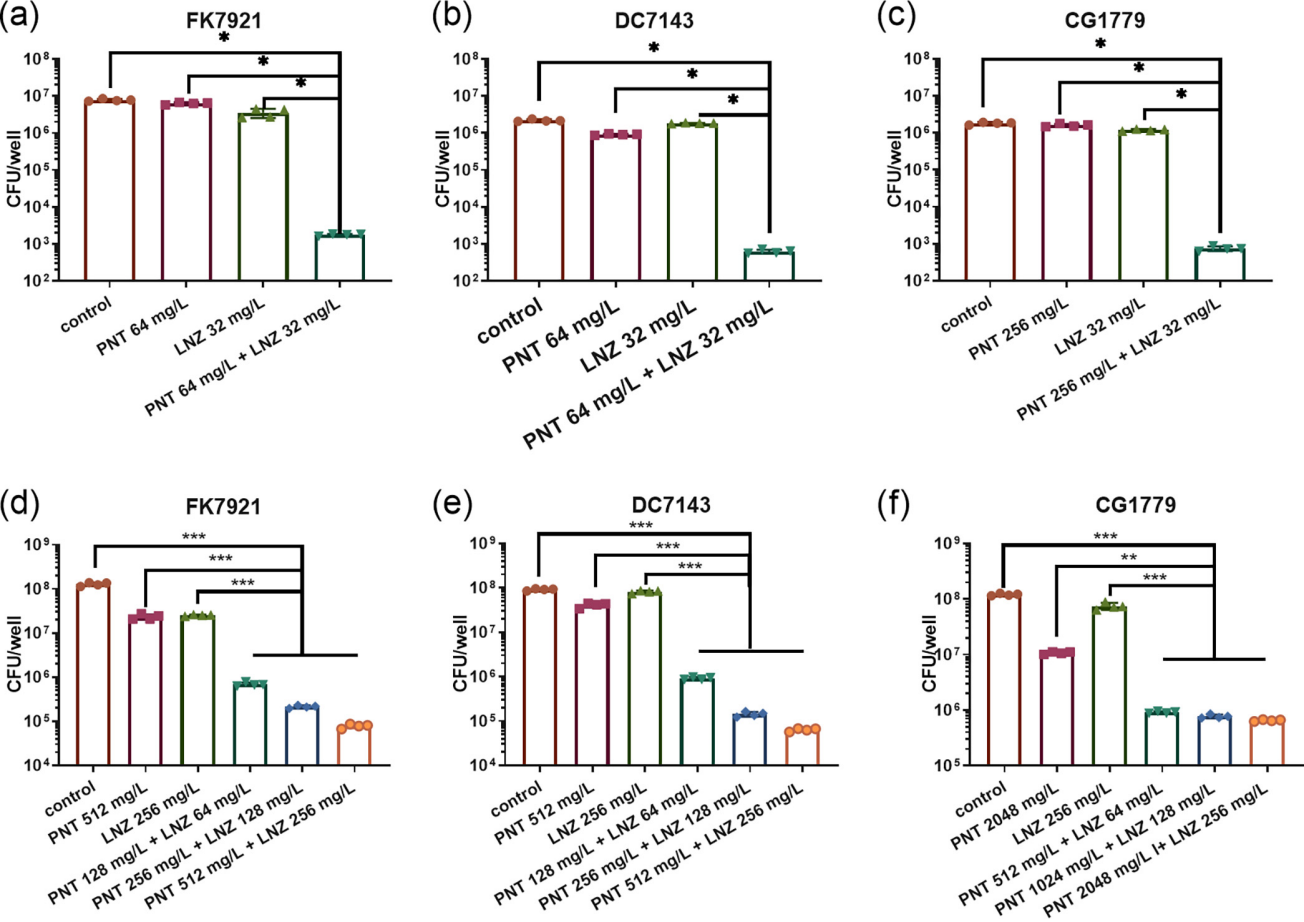
CFU analysis of the viable bacteria counts of the biofilms. Biofilms that are inhibitory under the pentamidine/linezolid combination or alone (1× MIC) in K. pneumoniae FK7921 (A), E. coli DC7143 (B), and E. cloacae CG1779 (C). Eradication of preformed biofilms with the treatment of a pentamidine/linezolid combination (2×, 4×, 8× MIC) or alone (8× MIC) in K. pneumoniae FK7921 (D), E. coli DC7143 (E), and E. cloacae CG1779 (F). The data are expressed as the mean ± standard deviation from four biological replicates. *, *P *< 0.05; **, *P *< 0.01; ***, *P *< 0.001, as analyzed via a Mann-Whitney test. PNT, pentamidine; LNZ, linezolid.

According to the colony forming units (CFU) analysis (Fig. 7), the pentamidine-linezolid combination reduced the bacterial loads of the CRE biofilms by about 3.36-log-fold, compared with that of pentamidine or linezolid alone (approximately 3.61-log versus approximately 0.16-log in K. pneumoniae FK7921 [Fig. 7a], approximately 3.63-log versus approximately 0.35-log in E. coli DC7143 [Fig. 7b], and approximately 3.42-log versus approximately 0.06-log in E. cloacae CG1779 [Fig. 7c]), indicating that the pentamidine-linezolid combination is a prospective therapeutic by which to inhibit the biofilm formation of CRE. The CFU analysis for the pentamidine-linezolid combination to eradicate the preformed biofilms of CRE showed that with the treatments of the 2×, 4×, and 8× MIC combinations, the bacterial loads decreased by about 1.35-log-fold to 3.04-log-fold, compared with that of pentamidine or linezolid alone (approximately 2.34-log, 2.85-log, and 3.31-log in the 2×, 4×, and 8× MIC combinations versus 0.71-log to 0.72-log in 8× MIC monotherapy in K. pneumoniae FK7921 [Fig. 7d]; approximately 1.62-log, 2.56-log, and 3.37-log versus 0.01-log to 0.62-log in E. coli DC7143, [Fig. 7e]; and approximately 2.52-log, 3.11-log, and 3.26-log versus 0.11-log to 1.1-log in E. cloacae CG1779, [Fig. 7f]), which indicated that the pentamidine**-**linezolid combinations had a robust ability to kill CRE in their preformed biofilms. Taken together, it can be concluded that the combination of the two drugs has a significant synergistic antibiofilm effect.

### *In vivo* synergy in the Galleria mellonella infection model.

**FIG 8:**
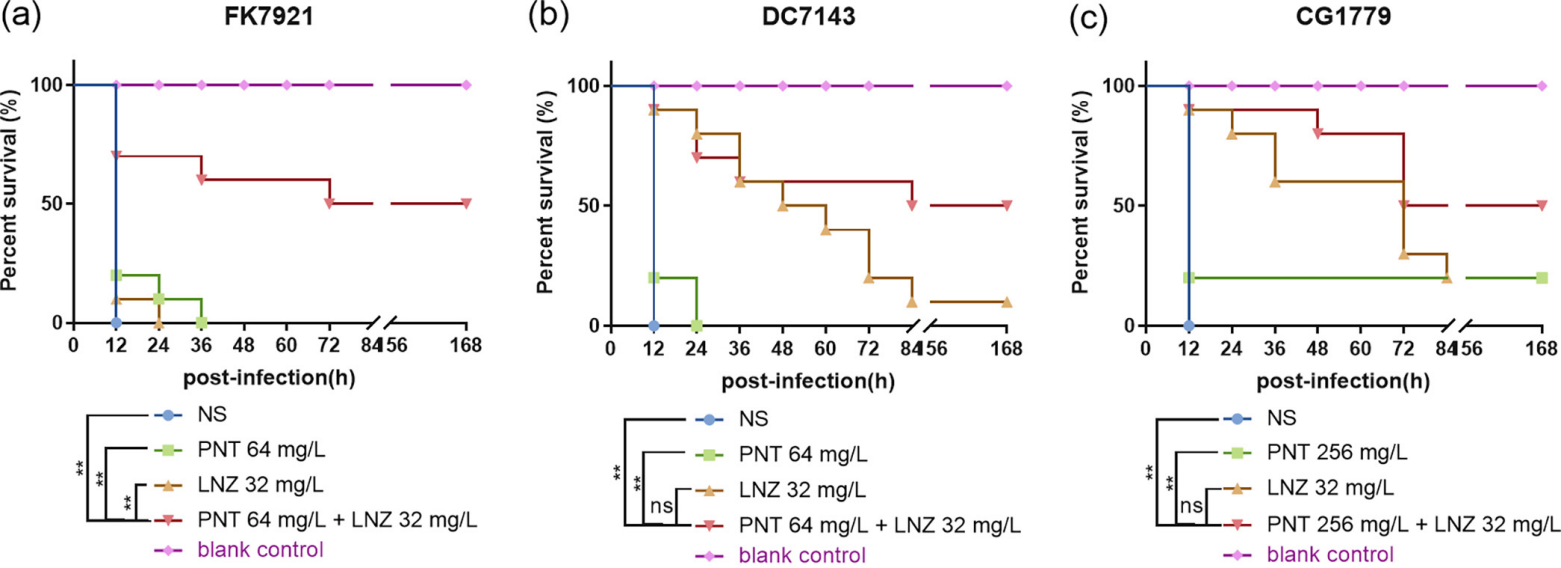
Therapeutic effects *in vivo* of a pentamidine-linezolid combination in the G. mellonella infection model. The differences in the survival rates of G. mellonella (10 larvae in each group) that were infected with 1.5 × 10^5^ CFU of 3 representative CRE isolates, namely, K. pneumoniae FK7921 (A), E. coli DC7143 (B), and E. cloacae CG1779 (C), within 168 h, with treatments of pentamidine and linezolid in combination or alone (red boxes in Fig. 1), were analyzed via a Kaplan-Meier analysis and a log-rank test. The NS groups represent the control conditions without any treatment but with the same volume of normal saline. No death of G. mellonella in the blank control groups (without infection but only NS injected) guaranteed the health and vitality of G. mellonella, with no human-induced injuries to it. ns, not statistically significant; *, *P *< 0.05; **, *P *< 0.01. PNT, pentamidine; LNZ, linezolid.

The *in vivo* efficacy of the pentamidine-linezolid combination against CRE infections was ascertained via G. mellonella survival experiments. As shown in Fig. 8, in the G. mellonella infection models of all three isolates, K. pneumoniae FK7921 (Fig. 8a), E. coli DC7143 (Fig. 8b), and E. cloacae CG1779 (Fig. 8c), the survival of worms was not observed within 12 h in the untreated control groups. Additionally, the linezolid monotherapy group also showed almost no therapeutic effect, with survival rates of only 20% in the first 12 h. However, treatments of pentamidine combined with linezolid maintained a survival rate of 50% at 168 h. The survival rates of G. mellonella with combination therapy were significantly increased, compared to those observed with linezolid monotherapy (*P *< 0.05) and the untreated condition (*P *< 0.05). Additionally, although there were no significant differences in the survival rates of G. mellonella between the pentamidine-linezolid pairs and the pentamidine monotherapy groups in E. coli DC7143 (Fig. 8b) or in E. cloacae CG1779 (Fig. 8c) (*P *> 0.05), those of the former were higher than those of the latter. So, we can conclude that the pentamidine-linezolid combination has a good therapeutic effect against CRE infections *in vivo*.

### *In vivo* efficacy assessment in mice.

**FIG 9:**
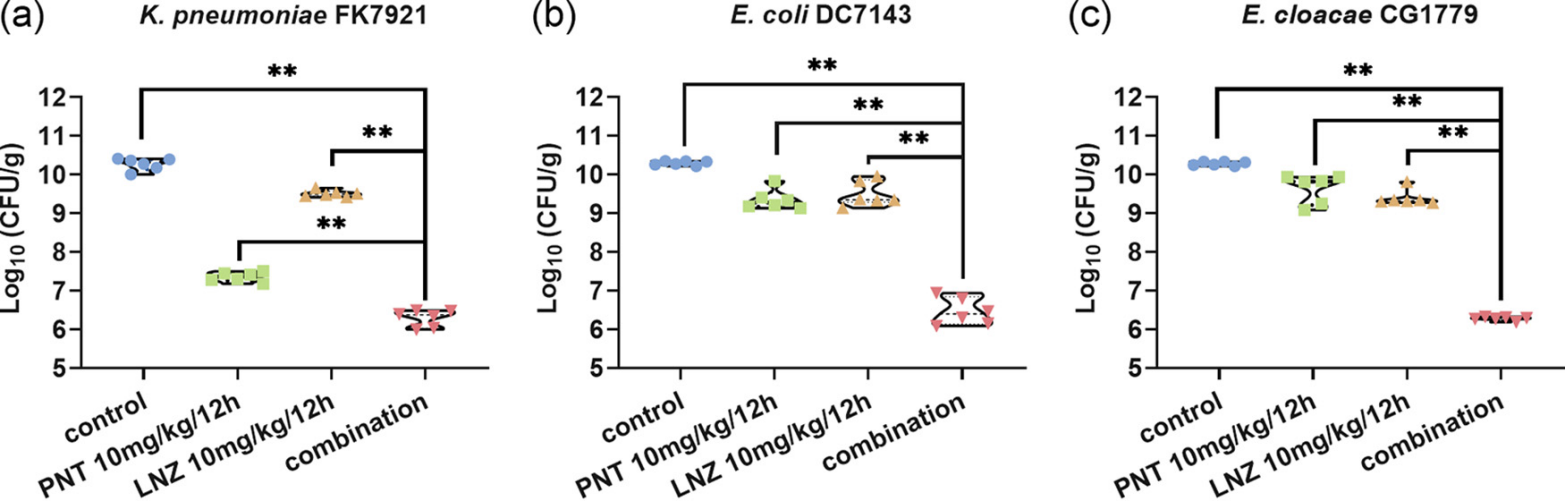
The *in vivo* efficacy of the pentamidine-linezolid combination in the mouse infection model. Log_10_ CFU/g_(thigh)_ in mice at 24 h posttreatment of monotherapy or combination therapy against carbapenem-resistant K. pneumoniae FK 7921 (A), E. coli DC7143 (B), and E. cloacae CG1779 (C) were quantified. Each point represents the mean of 3 repeat values for 1 thigh of 1 mouse (*n* = 6). **, *P *< 0.01. The statistical significance was analyzed via a Mann-Whitney test. PNT, pentamidine; LNZ, linezolid.

The *in vivo* efficacy of pentamidine in combination with linezolid was further validated by a mouse thigh infection model that was infected with carbapenem-resistant K. pneumoniae FK7921 (Fig. 9a), E. coli DC7143 (Fig. 9b), and E. cloacae CG1779 (Fig. 9c). As shown in Fig. 9, pentamidine or linezolid monotherapy lightly inhibited the load of CFU in the thigh muscles of the mice, compared with the untreated group. However, their combination showed a more dramatic decrease in CFU than did those of the groups treated with single agents (*P *< 0.05). These results indicate that the combination of the two drugs has a significant synergistic antibacterial efficacy against CRE infections *in vivo*.

### Mechanisms for drug synergy.

**FIG 10:**
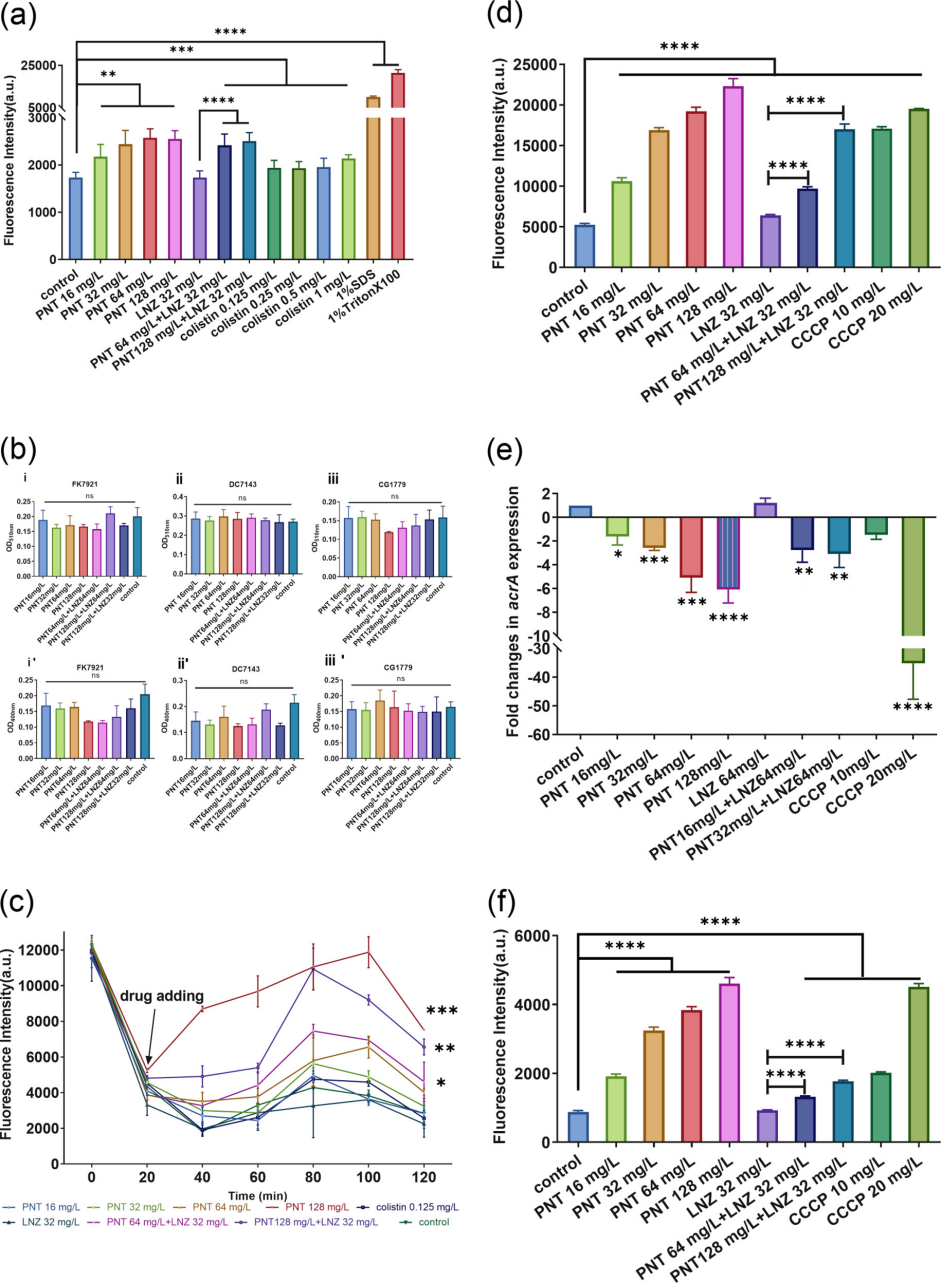
Mechanisms for the synergy of pentamidine and linezolid. (a) N-phenyl-1-naphthylamine (NPN) uptake assay, measuring the outer membrane permeability of FK7921 with the treatment of pentamidine, linezolid, and pentamidine-linezolid pairs with different concentrations. The well-documented outer membrane inhibitors colistin, SDS, and Triton X-100 were used as positive controls. The fluorescence intensity increased with the increasing concentration of pentamidine, indicating that pentamidine increased the outer membrane permeability in a dose-dependent manner. (b) Bacterial intracellular alkaline phosphatase (i, ii, iii) and β-1,4-galactanase (i′, ii′, iii′) leakage experiments. The absorbance values correspond to the amount of enzyme released extracellularly. There were no differences in the amount of enzyme released between the drug treatment and the drug-free control groups. (c) The fluorescence probe 3,3-dipropylthiadicarbocyanine iodide (DiSC_3_(5)) detects the membrane potential of K. pneumoniae FK7921. The fluorescence intensity of DiSC_3_(5) in the culture medium was monitored to measure the effect on the bacterial membrane potential of different concentrations of pentamidine and linezolid in monotherapy or pentamidine and linezolid in combination with sub-MIC colistin (0.125 mg/L) as a positive control. The more pronounced the effect of the drug in reducing the membrane potential, the higher the fluorescence value of DiSC_3_(5). (d) Intracellular accumulation of acridine orange (AO) in K. pneumoniae FK7921 treated with different concentrations of pentamidine and linezolid in monotherapy or pentamidine and linezolid in combination. AO is a recognized substrate of the bacterial efflux pump. With the inhibition or loss of the function of the bacterial efflux pump, AO penetrates the bacterium and binds to its DNA, thereby producing fluorescence. The fluorescence intensity of the intracellular AO represents the extent of the damage to the efflux pump. (e) The expression of the efflux pump gene *acrA*, which reflexes the effects of drugs on the efflux pump, was determined via qPCR in K. pneumoniae FK7921 treated with different concentrations of pentamidine and linezolid in monotherapy or pentamidine and linezolid in combination. Pentamidine significantly reduced the gene expression levels in a dose-dependent manner. (f) 2′,7′-dichlorodihydrofluorescein diacetate (H2DCFDA) detecting the ROS of K. pneumoniae FK7921. The standard efflux pump inhibitor CCCP was used as a positive control (panels D, E and F). ns, not statistically significant; *, *P *< 0.05; **, *P *< 0.01. ***, *P *< 0.001; ****, *P *< 0.0001, as analyzed via a Student’s *t* test. PNT, pentamidine; LNZ, linezolid.

Outer membrane impermeability appears to be the main contributor to the intrinsic resistance of GNB to Gram-positive antimicrobials (8). However, under the synergistic effect of pentamidine, linezolid became active in GNB. We hypothesize that pentamidine might specifically inhibit or destroy the outer membrane of GNB and thereby assist linezolid in its entry into GNB cells to achieve effective accumulation and bind to its targets to exert an antibacterial effect. Therefore, we first measured the effect of pentamidine on the outer membrane permeability in the representative K. pneumoniae FK7921 strain via a 1-N-phenylnaphthylamine (NPN) uptake assay. As expected, pentamidine increased the outer membrane permeability of FK7921 in a dose-dependent manner, whereas linezolid monotherapy did not (Fig. 10a). To determine the extent to which pentamidine disrupted the outer membrane, we performed intracellular enzyme leakage experiments on strains, including K. pneumoniae FK7921, E. coli DC7143, and E. cloacae CG1779. This set was considered to contain a representative strain of each species. Surprisingly, there were no differences in the amounts of alkaline phosphatase (Fig. 10b, panels i, ii, and iii) or β-1,4-galactanase (Fig. 10b, panels i′, ii′, iii′) that were released between the drug treatment and the drug-free control groups, indicating that pentamidine may not cause serious structural damage to the GNB membrane, which is not enough to cause the random leakage of macromolecular substances with representative substances as intracellular enzymes. As such, we investigated whether pentamidine disrupted the charge barrier of the membrane. As shown in Fig. 10c, high concentrations of pentamidine significantly dissipated the membrane potential of K. pneumoniae FK7921. It has been reported that linezolid is a substrate of the efflux pump of GNB, which is proposed as another reason why GNB is naturally resistant to linezolid (14). The addition of sub-MICs of the efflux pump inhibitor carbonyl cyanide m-chlorophenylhydrazone (CCCP) significantly decreased the MIC values of linezolid against CRE strains, including K. pneumoniae FK7921, E. coli DC7143, and E. cloacae CG1779 (Table 2). The efflux pump inhibitor resensitized GNB to linezolid, indicating that the resistance to linezolid in GNB is indeed mainly mediated by the efflux pump and that pentamidine in the combination may act as an efflux pump inhibitor, thereby potentiating the effect of linezolid in GNB. Acridine orange (AO) is a recognized substrate of the bacterial efflux pump, and it usually cannot enter the bacterial cells when the efflux pumps function normally (7). The elevated intracellular accumulation of AO with the increased concentration of pentamidine demonstrated that pentamidine is a potential efflux pump inhibitor (Fig. 10d). Similarly, the expression of the efflux pump gene *acrA*, which reflexes the effects of drugs on the efflux pump, showed that pentamidine significantly reduced the gene expression levels in a dose-dependent manner (Fig. 10e). In addition, it was found that pentamidine significantly enhanced the production of intracellular reactive oxygen species (ROS) in a dose-dependent manner but that linezolid monotherapy did not (Fig. 10f). These results indicated that pentamidine possibly worked as an adjuvant, potentiated the rapid accumulation of linezolid and ROS in GNB cells, and eventually killed the GNB cells by inhibiting their outer membranes, membrane potentials, and efflux pumps.

**TABLE 2:** MICs of linezolid against GNB with or without CCCP

Strain	MIC (mg/L)		
	Linezolid^*a*^	Linezolid + 10 mg/L CCCP^*a*^	CCCP^*b*^
K. Pneumoniae FK7921	256	<0.5	32
E. coli DC7143	1,024	<0.5	64
E. cloacae CG1779	1,024	<0.5	32

a MIC of linezolid.

b MIC of CCCP.

## DISCUSSION

Given the particularly alarming emergence and spread of CRE, new strategies by which to address the rapidly diminishing number of treatment options for their relevant infections are needed (3). Here, we introduced a novel drug combination strategy against these problem pathogens. That is, the otherwise ineffective, GPB antimicrobial linezolid becomes active in GNB when combined with an adjuvant-pentamidine. We evaluated their synergistic antibacterial and antibiofilm effects *in vitro* as well as their efficacy *in vivo* in G. mellonella and mouse infection models against CRE. Even more significantly, we elucidated the potential mechanisms for the synergy of the combination.

First, a checkerboard assay showed significant synergistic antibacterial effects, *in vitro*, of the combination against clinical CRE isolates. Specifically, pentamidine alone showed high MICs, ranging from 256 to 2,048 mg/L, indicating that its monotherapy is ineffective against CRE, which was consistent with the results reported in other studies, which demonstrated a range from 100 to 1,600 mg/L against CRE or P. aeruginosa (5, 12, 13). Similarly, for linezolid, the MICs of its single agent, ranging from 512 to ≥1,024 mg/L, were in keeping with its almost native resistance by GNB. However, when combined, the MICs of pentamidine were dramatically decreased by 16 to 128-fold and linezolid by 8 to >64-fold (Fig. 1), which indicated that their combination has exceptional synergistic antibacterial activity against CRE. The time-kill assay further proved this effect in dynamic conditions meant to simulate the clinic. The combination of pentamidine and linezolid showed remarkable bactericidal activities within 12 h as well as good synergistic bacteriostatic effects that lasted for up to 24 h against CRE (Fig. 3). These data were not in line with the results of a study in which pentamidine alone at MICs showed bactericidal activity *in vitro* against CRE (12). In particular, our checkerboard experiment showed no synergistic effect for the carbapenem and colistin-coresistant E. coli isolate, which was in concordance with the finding that pentamidine alone showed no enhanced activity against colistin-resistant E. coli (5).

Furthermore, we selected one of the effective synergistic concentrations of the pentamidine-linezolid combination (as indicated by the red boxes in Fig. 1) for each tested strain, in which the concentrations of linezolid, showing no cytotoxicity (Fig. 2), were significantly lower than the intrapulmonary concentrations for the recommended dosage regimen and thus are achievable in patients (15–18). Similarly, 32 to 256 mg/L pentamidine was selected, as it was assessed as relatively safe in cell and animal experiments (Fig. 2). In addition, pentamidine, when used as an aerosol, is selectively distributed to the lungs without systemic side effects, even at a comparatively high dose (19), and an important application of linezolid is the treatment of GPB-associated pulmonary infections (20). Their combination is expected to be designed as an aerosol to treat pulmonary infections. In this way, they may be relatively safe for further clinical applications. More encouragingly, with pentamidine as an antiprotozoal and antifungal agent (21) and linezolid as a defensive GPB drug (6), their combination expanding the treatment range to GNB may be an effective treatment of complex infections that are mixed by GPB, GNB, and even fungi.

One of the challenges associated with CRE is their biofilms, which render more resistant, recurrent, or even incurable infections (2). Thus, controlling the biofilm-forming bacteria is crucial in combating biofilm formation. This is the first study to investigate the potential utility of a pentamidine-linezolid combination to treat CRE-associated biofilm formation at an early stage and to also eradicate mature biofilms at a later stage. Their combination could inhibit CRE biofilm formation (Fig. 4), and similar results are also supported by the SEM visualization (Fig. 5) and viable bacteria counting of the biofilms (Fig. 7). Considering that the disruption of preformed biofilms is more difficult than biofilm inhibition (22), we used 2×, 4×, and 8× combination concentrations against mature biofilms that were formed at 24 h. The results demonstrated a significantly synergistic and concentration-dependent biofilm eradication effect of pentamidine-linezolid (Fig. 6 and 7). Collectively, these results are encouraging, and they suggest that pentamidine-linezolid could be further used as a promising antibiofilm agent.

Moreover, we established the G. mellonella and mouse infection models with representative CRE strains, and this was followed by treatments with different concentrations of the drugs to confirm the *in vivo* efficacy of pentamidine-linezolid against CRE. Our data showed the successful control of CRE-relevant infections *in vivo* using the combination (Fig. 8 and 9), which is similar to the finding that pentamidine combined with GPB antimicrobials exhibited synergistic activity against systemic A. baumannii infections in mice (5).

Finally, we explored the underlying mechanism by which the two drugs exerted their synergistic anti-CRE effect. On the one hand, pentamidine increased the outer membrane permeability of GNB by disrupting the charge barrier of the membrane. The activity of efflux pumps is known to be maintained by the proton motive force (23). The dissipation of the membrane potential induces the dysfunction of the efflux pump, thereby allowing the intracellular accumulation of the substrate of the efflux pump. Subsequently, the accumulation of ROS in bacterial cells, due to the proton motive force dissipation, reduces ATP production, impairs the respiration chain, and induces the accumulation of intracellular antibiotics, which ultimately results in the death of the bacteria (24). On the other hand, these functions are only observed as being exercised by pentamidine, not linezolid (Fig. 10), indicating that pentamidine probably acts as an adjuvant and helps linezolid to rapidly accumulate in a GNB cell and eventually kill it. Notably, pentamidine cannot cause serious structural damage to the outer membrane, and it can only act at the level of charge. This effect may have a certain selectivity to the partner drugs with which pentamidine can combine, and it may be weaker than that due to the substantial disruption of the outer membrane structures, which may explain why the concentrations for the synergy of the two drugs are somewhat high.

In summary, to our knowledge, this is the first study that introduces a new combination of pentamidine as an antimicrobial adjuvant with GPB antimicrobial linezolid into GNB treatment. It poses potential *in vitro* synergistic antibacterial and antibiofilm activity as well as *in vivo* efficacy against CRE. In combination, pentamidine acts as a potential inhibitor of the outer membrane and the efflux pump of GNB, and it resensitizes GNB to linezolid. Despite the shortcoming of relatively high combination concentrations, our work might contribute to the provision of a possible research direction for the repurposing of old drugs with the adjuvant potential to expand the therapeutic range of existing drugs. Perhaps the use of pentamidine to enhance linezolid against CRE is an extreme example, but pentamidine may be easier to enhance other drugs or against other bacteria, which we can explore further. It is encouraging that their combination is expected to be designed as an aerosol to treat pulmonary infections, especially for the emerging mixed infections of GPB, GNB, and even fungi. In this way, they might be relatively safe for further clinical applications. Taken together, we believe that pentamidine is still a potential antibacterial adjuvant or sensitizer, especially in the face of the negative situation of severe MDR bacterial infections as well as the slow and costly development of new drugs.

## MATERIALS AND METHODS

### Drugs and dissolvent reagents.

Pentamidine (MedChemExpress Co., Ltd., USA) was initially dissolved in dimethyl sulfoxide (DMSO) (Sigma-Aldrich, USA) (final concentration of DMSO ≤2.5% [vol/vol]) (25). Linezolid (Kangtai, Wenzhou, China) was initially dissolved in sterile distilled water and was subsequently dissolved in CAMHB (Sigma-Aldrich, USA) to obtain the desired drug concentrations.

### Bacterial strains and antimicrobial susceptibility testing (AST).

A total of 10 nonduplicated Gram-negative clinical isolates, including E. coli (*n* = 4), K. pneumoniae (*n* = 3), and E. cloacae (*n* = 3), were recovered from a tertiary teaching hospital (Wenzhou, China). All of the strains were identified to the species level via matrix-assisted laser desorption/ionization time-of-flight mass spectrometry (MALDI-TOF/MS; bioMérieux, France). Routine AST was done using a bioMérieux Vitek-2 (bioMérieux, France), and the results were interpreted using the CLSI guidelines (M100-S31, 2021) (26). The MICs of pentamidine and linezolid, without defined breakpoints against GNB in CLSI, were determined via broth microdilution. E. coli ATCC 25922 and P. aeruginosa ATCC 27853 (the National Center of Clinical Laboratory [NCCL]) served as the quality controls.

### Checkerboard assay.

The *in vitro* synergistic antimicrobial activity of the pentamidine-linezolid combination against CRE was examined via a checkerboard assay (4). 50 μL of pentamidine (2 to 2,048 mg/L) were set along the *x* axis, 50 μL of linezolid (16 to 1,024 mg/L) were set along the *y* axis, and 50 μL of pentamidine or linezolid alone plus 50 μL of CAMHB were set along the bottom row and the most left column, respectively, to obtain 100 μL of drug-containing medium in a 96-well plate (Corning, USA). Then, 100 μL of the bacterial suspension (10^6^ CFU/mL) were added to each well. The MICs of the single drugs and the combination were recorded after 18 to 24 h. The fractional inhibitory concentration index (FICI) values were calculated as FICI = (MIC drug A in combination) **/** (MIC drug A) + (MIC drug B in combination) **/** (MIC drug B) (14). Synergy was defined as FICI ≤ 0.5, with no interaction being defined as 0.5< FICI ≤ 4 and antagonism being defined as FICI > 4 (27).

### Cytotoxicity assay.

The cytotoxicities of pentamidine, linezolid, their combination, and 2.5% (vol/vol) DMSO, were tested on RAW264.7 cells (Procell Life Science and Technology Co., Ltd., China) via the Cell Counting Kit-8 (CCK-8) method. 2 × 10^5^ cell lines were seeded at 100 µL/well into 96-well tissue culture plates (Corning, USA) and incubated for 12 h. Following treatment with different concentrations of pentamidine and/or linezolid as well as 2.5% (vol/vol) DMSO (the control group with cells only), the cell lines were incubated in the presence of CO_2_ at 37°C for 24 h. After that, 10 µL of CCK-8 reagent (MedChemExpress, USA) were added and incubated for 2 h at 37°C. The absorbance was measured at 450 nm using a microplate reader (Multiskan FC).

### Hemolytic activity assay.

We further verified the effects of pentamidine, linezolid, and their combination on mouse RBCs, as described elsewhere (28). Briefly, fresh blood from healthy mice was collected and centrifuged to remove the plasma and mononuclear cells. The Laboratory Animal Ethics Committee of the First Affiliated Hospital of Wenzhou Medical University approved these analyses (WYYY-AEC-2022-048) and was consistent with the Chinese National Standards for Laboratory Animals (GB 14925–2010). The RBCs were washed three times with normal saline (NS) and were diluted with NS to a final concentration of 5%. 400 μL of different concentrations of the tested drugs were added to 2 mL of a 5% RBC suspension. After incubation at 37°C for 1 h and centrifugation at 3,000 rpm for 5 min, the absorbance of the supernatant at 540 nm was measured to reflect hemolysis. RBCs treated with NS and 0.1% (vol/vol) Triton X-100 served as the negative and positive controls, respectively. Hemolysis rate (%) = (OD_experimental group_ − OD_NS_) / (OD_0.1% [vol/vol] Triton X-100_ − OD_NS_).

### Time-kill assay.

A time-kill assay was conducted on six randomly selected CRE isolates (two K. pneumoniae, two E. coli, and two E. cloacae) (9). In brief, 200 μL of a 0.5 McFarland bacterial suspension were added to 20 mL of fresh LB broth (Oxoid, Britain) for a 100-fold dilution in order to obtain an initial concentration of approximately 10^6^ CFU/mL. The selected combination and corresponding single-agent concentrations, as per the checkerboards, were set as combination therapy groups and monotherapy groups, whereas the control groups were treated without drugs but with 2.5% (vol/vol) DMSO. Viable cell counting was performed on the LB agar plates at 0, 2, 4, 6, 12, and 24 h. The bactericidal activity was defined as a ≥3 log_10_ CFU/mL decrease, compared with the initial bacterial concentration. The synergy was considered if the reduction in CFU/mL of the combination was ≥2 log_10_, compared with the most active monotherapy at 24 h (9).

### Biofilm formation and eradication assays.

Both the CV (22) and colony-forming unit (CFU) plate counting methods (29) were used to assess the antibiofilm efficacy, including the biofilm formation and eradication, of pentamidine and linezolid, alone or in combination. The difference between the biofilm formation assay (0, 1× MIC) and the eradication assays (0, 2×, 4×, 8× MIC) was that the drugs were added simultaneously with the bacteria in the former, whereas in the latter, the drugs were added after the bacterial biofilm had formed for 24 h. The stock solutions of drugs were diluted to 2-fold or 4-fold of the desired final concentration in fresh LB broth. Then, 100 μL 2× (monotherapy group) or 50 μL 4× (combination group) drug diluent, which was replaced by 100 μL LB broth in the control groups, plus 100 μL diluted bacterial suspension were transferred to the wells of a 96-well microtiter plate (Corning, USA). The wells were washed after 24 h to remove the planktonic bacteria. For the biofilm mass determination, 0.1% (wt/vol) CV (Solarbio, China) was used to stain the biofilms, and the OD_570nm_ was measured using a microplate reader (Multiskan FC) to obtain the biofilm mass. For the CFU analysis of the viable bacteria counts of the biofilms, the biofilms were harvested via scraping with a sterile pipette tip, and this was followed by sonication and vortexing. After serial dilutions, cells from the biofilms were plated on LB plates. The viable cell density was calculated to determine the antimicrobial effect against the biofilms. Both CV assays and CFU plate counting assays were performed in biological triplicates.

### Scanning electron microscope (SEM).

SEM was used to further study the biofilm inhibitory effect of the combination. FK7921, DC7143, and CG1779 (i.e., one representative strain from each of the three species) were selected for SEM. They were, respectively, treated with or without linezolid, pentamidine, or their combination for 24 h on sterile cell climbing slices (CITOGLAS, China) in a 6-well plate (NEST, China). After the treatments, the cell climbing slices were rinsed with sterile PBS three times and were then fixed with 2.5% (vol/vol) glutaraldehyde (Aladdin, China) at 4°C overnight. After drying, the samples were dehydrated by 20%, 40%, 70%, 90%, 95%, and 100% (vol/vol) ethanol. Finally, the samples were gold-sputtered and observed via SEM (S-3000N, Japan).

### G. mellonella *in vivo* assays.

G. mellonella and its infection model were used to evaluate the toxicity and synergistic antimicrobial effects of the drugs *in vivo*. For the *in vivo* safety evaluation of the drugs, 10 μL of different concentrations of pentamidine were injected into healthy larvae (10 worms in each group). This was followed by the observation of their postinjection survival rates for 7 days. The concentration that resulted in death for more than half of the larvae (i.e., the 50% lethal dose [LD_50_]) was considered to be toxic *in vivo*. For the *in vivo* treatment effects of the drugs, the survival curves at 7 days postinfection under different drug-treatment conditions were compared. 10 μL (1.5 × 10^5^ CFUs) of a bacterial suspension of FK7921, DC7143, or CG1779 was injected into G. mellonella (10 worms in each group). This was followed by treatments at 2 h postinfection with 10 μL of drugs of 7× MICs in combination (pentamidine 64 mg/L + linezolid 32 mg/L, pentamidine 256 mg/L + linezolid 32 mg/L ×7) or alone (pentamidine 64, 256 mg/L × 7; linezolid 32 mg/L × 7), whereas the control groups (with infection but without treatment) were treated with the same volume of NS. The blank control groups (without infection and only NS injected) were set up to control the health and vitality of the G. mellonella as well as the human-induced injury to it. The survival rates of G. mellonella were recorded every 12 h until 168 h by observing their skin color and response to stimuli.

### Mouse infection model.

All of the animal studies were approved by the Laboratory Animal Ethics Committee of the First Affiliated Hospital of Wenzhou Medical University (WYYY-AEC-2022-048) and were conducted according to the Guidelines for the Ethical Review of the Welfare of Laboratory Animals. A neutropenic mouse thigh infection model was used for the *in vivo* efficacy studies. In short, a total of 36 female BALB/c mice, aged 5 to 6 weeks (Vital River Laboratory Animal Technology Co., Ltd., Zhejiang, China), were intraperitoneally injected with cyclophosphamide (Yuanye Biotechnology Co., Ltd., Shanghai, China) for 3 days (150 mg/kg) or for 1 day (100 mg/kg) to induce a neutropenia model before infection. 12 mice were challenged with the same CFU of one isolate (1.5 × 10^7^ CFU of K. pneumoniae FK7921, 8 × 10^7^ CFU of E. coli DC7143, or 6 × 10^7^ CFU of E. cloacae CG1779) in each posterior thigh muscle for 2 h. The 12 mice were randomly divided into 4 groups (3 mice [5] and 6 thighs for each group; the sample size was selected based on the results of a preliminary infection trial) and were intraperitoneally administered with pentamidine (10 mg/kg), linezolid (10 mg/kg), their combination (10 mg/kg pentamidine + 10 mg/kg linezolid) (9), or PBS as a control. The treatments were further added at 12 h postinfection. The mice were euthanized at 24 h after treatment, and the bacterial burden was quantified based on the CFU counts from the posterior thigh homogenates.

### Outer membrane permeability assay.

The outer membrane permeability was measured using a hydrophobic fluorescent probe 1-N-phenylnaphthylamine (NPN, Aladdin, Shanghai, China) uptake method, as previously described (24), with some modifications. K. pneumoniae FK7921 was grown to the mid-logarithmic phase with an OD of 0.4 to 0.6 at 600 nm in LB. Equal volumes of bacterial solution were allocated to the designated tubes for the experimental groups with the desired concentrations of pentamidine monotherapy, linezolid monotherapy, or combination therapy as well as for the positive-control groups with colistin (Kangtai, Zhejiang, China), SDS (Solarbio, China), and Triton X-100 (Solarbio, China) and the negative-control group, which contained only the bacteria. The tubes were incubated at 37°C with shaking at 180 rpm for 2 h. The bacterial solutions were then pelleted and washed three times in PBS buffer (pH 7.2) with 5 mM glucose and were subsequently resuspended with 1 mL of 10 μM NPN working solution. 200 μL of the resulting cell suspension was added to each well of a black 96-well plate. After incubation at room temperature for 30 min in darkness, a multimode plate reader (VICTOR Nivo, PerkinElmer, USA) was used to measure the fluorescence at an excitation wavelength and an emission wavelength of 350 and 420 nm, respectively.

### Intracellular enzyme leakage assays.

For each overnight-grown culture of FK7921, DC7143, and CG1779, equal volumes of bacterial solution were allocated to the designated tubes with four different treatment conditions: blank control, linezolid alone, pentamidine alone, and their combination. The tubes were incubated at 37°C with shaking at 180 rpm for 24 h. Subsequently, the supernatant was collected via centrifugation, and the activities of alkaline phosphatase and β-1,4-galactanase in the supernatant were determined using the respective commercial kits (Solarbio, China).

### Membrane potential assay.

The membrane potential was detected using a fluorescence probe 3,3-dipropylthiadicarbocyanine iodide (DiSC_3_(5), Aladdin, Shanghai, China), as previously described (24), with some modifications. Overnight-cultured K. pneumoniae FK7921 cells were collected via centrifugation and were washed with PBS (pH 7.2). Then, the cells were resuspended in a 30 μM DiSC_3_(5) working solution. The background fluorescence intensity was monitored at an excitation/emission of 622/670 nm until 20 min, at which time the indicated concentration of pentamidine monotherapy, linezolid monotherapy, combination therapy, or sub-MIC colistin (0.125 mg/L) as a positive control was added to the cell suspension. The fluorescence was continuously monitored for 120 min.

### Effect of CCCP on the MICs of linezolid.

The broth microdilution (BMD) method was used for the determination of the MICs of linezolid in the absence and presence of sub-MIC CCCP (MedChemExpress Co., Ltd., USA) against FK7921, DC7143, and CG1779 by using CAMHB (Sigma-Aldrich, USA). The MICs of the isolates to CCCP were predetermined using BMD. The concentration of linezolid that was used per well was serially increased by 2-fold, whereas that of CCCP was kept constant at a final concentration of 10 mg/L.

### Acridine orange (AO) intracellular accumulation assay.

An aliquot of bacterial solution in the mid-logarithmic phase was allocated to the designated tubes: (i) in the presence of pentamidine and linezolid, alone or in combination; (ii) in the presence of subinhibitory concentrations of CCCP (10 and 20 mg/L); and (iii) under drug-free conditions. The tubes were incubated at 37°C with shaking at 180 rpm for 12 h. The bacterial solutions were then pelleted and washed three times with PBS buffer (pH 7.2) and were subsequently resuspended with 1 mL of 30 μM AO (MedChemExpress Co., Ltd., USA) working solution. 200 μL of the resulting cell suspension were added to each well of a black 96-well plate. After incubation at room temperature for 1 h in darkness, the fluorescence at an excitation wavelength and an emission wavelength of 488 and 515 nm, respectively, was measured using a multimode plate reader (VICTOR Nivo, PerkinElmer, USA).

### Quantitative real-time PCR (RT-qPCR) analysis.

To analyze the effect of pentamidine on the expression of the efflux pump-related gene *acrA*, an aliquot of 50 μL of overnight K. pneumoniae FK7921 culture was added to 5 mL of fresh LB broth supplemented with different concentrations of pentamidine and linezolid, alone or in combination (experimental groups), CCCP (positive control groups) or no drug (negative control group). The tubes were incubated at 37°C with shaking at 180 rpm for 24 h. Subsequently, the total RNA was extracted from the bacterial pellet cells via the TRIzol method, and the gene expression was determined using the SYBR Green (TaKaRa, Dalian, China) method with an RT-qPCR detection system. The *ropB* gene was used as the internal control.

### Reactive oxygen species (ROS) detection.

The bacterial intracellular ROS levels were detected using a 2′,7′-dichlorodihydrofluorescein diacetate (H2DCFDA) (Solarbio, China). Equal volumes of bacterial cultures of K. pneumoniae FK7921 were treated with pentamidine**-**linezolid or a sub-MIC of CCCP as a positive control at the indicated concentrations. Then, they were incubated at 37°C with shaking at 180 rpm for 2 h. The bacterial solutions were then pelleted and washed with PBS buffer (pH 7.2) and were subsequently resuspended with 1 mL of 30 μM H2DCFDA working solution. 200 μL of the resulting cell suspension were added to each well of a black 96-well plate. After incubation at room temperature for 1 h in darkness, a multimode plate reader (VICTOR Nivo, PerkinElmer, USA) was used to measure the fluorescence at an excitation wavelength and an emission wavelength of 488 and 535 nm, respectively.

### Statistical analysis.

Each experiment was performed in triplicate. Statistical significance was determined via a two-sample *t* test, a Mann-Whitney test, or a one-way analysis of variance (ANOVA). The survival of G. mellonella larvae was analyzed via a Kaplan-Meier analysis and a log-rank test. *, *P *< 0.05; **, *P *< 0.01; ***, *P *< 0.001; and ****, *P *< 0.0001. The statistical analyses were performed using the GraphPad Prism 8.03 software package.

### Data availability.

The data sets that were generated for this study are available upon request to the corresponding author.
